# Retargeting of T lymphocytes to PSCA- or PSMA positive prostate cancer cells using the novel modular chimeric antigen receptor platform technology “UniCAR”

**DOI:** 10.18632/oncotarget.15572

**Published:** 2017-02-21

**Authors:** Anja Feldmann, Claudia Arndt, Ralf Bergmann, Simon Loff, Marc Cartellieri, Dominik Bachmann, Roberta Aliperta, Mirjam Hetzenecker, Florian Ludwig, Susann Albert, Pauline Ziller-Walter, Alexandra Kegler, Stefanie Koristka, Sebastian Gärtner, Marc Schmitz, Armin Ehninger, Gerhard Ehninger, Jens Pietzsch, Jörg Steinbach, Michael Bachmann

**Affiliations:** ^1^ Helmholtz-Zentrum Dresden-Rossendorf (HZDR), Institute of Radiopharmaceutical Cancer Research, Dresden, Germany; ^2^ UniversityCancerCenter (UCC) ‘Carl Gustav Carus’ TU Dresden, Tumor Immunology, Dresden, Germany; ^3^ GEMoaB Monoclonals GmbH, Dresden, Germany; ^4^ Cellex Patient Treatment GmbH, Dresden, Germany; ^5^ Institute of Immunology, ‘Carl Gustav Carus’, TU Dresden, Dresden, Germany; ^6^ Medical Clinic and Policlinic I, University Hospital ‘Carl Gustav Carus’, TU Dresden, Dresden, Germany; ^7^ German Cancer Consortium (DKTK), partner site Dresden; and German Cancer Research Center (DKFZ), Heidelberg, Germany; ^8^ National Center for Tumor Diseases (NCT), Dresden, ‘Carl Gustav Carus’ TU Dresden, Dresden, Germany; ^9^ Department of Chemistry and Food Chemistry, School of Science, TU Dresden, Dresden, Germany

**Keywords:** CAR, retargeting, T cells

## Abstract

New treatment options especially of solid tumors including for metastasized prostate cancer (PCa) are urgently needed. Recent treatments of leukemias with chimeric antigen receptors (CARs) underline their impressive therapeutic potential. However CARs currently applied in the clinics cannot be repeatedly turned on and off potentially leading to severe life threatening side effects. To overcome these problems, we recently described a modular CAR technology termed UniCAR: UniCAR T cells are inert but can be turned on by application of one or multiple target modules (TMs). Here we present preclinical data summarizing the retargeting of UniCAR T cells to PCa cells using TMs directed to prostate stem cell- (PSCA) or/and prostate specific membrane antigen (PSMA). In the presence of the respective TM(s), we see a highly efficient target-specific and target-dependent activation of UniCAR T cells, secretion of pro-inflammatory cytokines, and PCa cell lysis both *in vitro* and experimental mice.

## INTRODUCTION

Prostate cancer (PCa) is still one of the leading cause of cancer-related death in men [1, 2]. While primary localized tumors may be manageable by prostatectomy or radiotherapy approximately 20% of patients develop metastatic disease within the following fifteen years [1, 3, 4]. Treatment options for such metastatic hormone-refractory tumors are rather limited underlining the need for innovative therapies [1–4].

**Figure 1:**
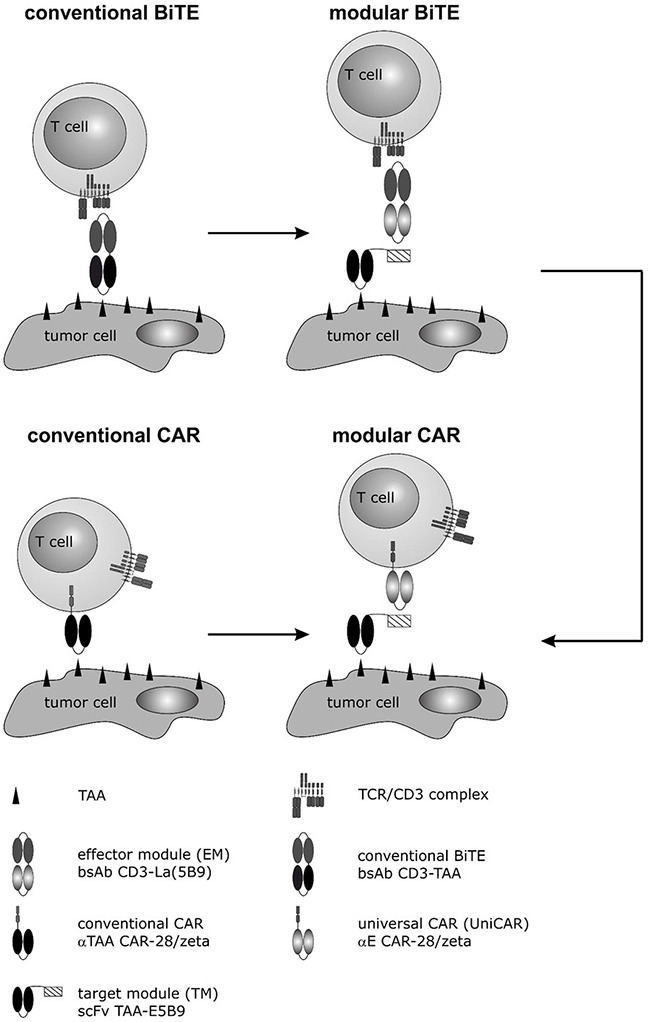
Development of a modular CAR against PSCA and PSMA for treatment of PCa cells In previous studies we described a mab directed to the prostate stem cell antigen PSCA [15]. Based on the variable domains of this mab a humanized bispecific Ab in a conventional BiTE format was established [14, 17]. Although highly active, to accelerate the development of future BiTEs we developed a novel modular BiTE format by splitting conventional BiTEs into two molecules: (i) a bispecific effector module (EM), which binds to the activating CD3 complex of T cells via one of its arms, and (ii) a tumor cell recognizing target module (TM) e.g. a scFv directed against PSCA or PSMA [16, 43]. The second arm of the EM is directed to a peptide epitope (E5B9). As this peptide epitope is fused to the TM, EM and TM can form a bispecific immune complex (modular BiTE) which was shown to mediate killing of tumor cells comparably well to conventional BiTEs [16, 18]. In parallel to conventional BiTEs, we used the same variable domains to establish conventional CARs directed to PSCA or PSMA [41, 43]. Moreover, based on the scFv domain of the EM directed to the peptide epitope (E5B9), we developed a second generation CAR as universal effector CAR module (modular CAR) which we termed Universal CAR (UniCAR) [46]. So far, we could already show “proof of concept” for retargeting of UniCAR T cells to leukemic cells (AML blasts) using TMs (tagged with the E5B9 epitope) directed to CD33 or CD123. In line with these studies, we now wanted to learn whether the TMs against PSCA and PSMA originally designed for the modular BiTE strategy [16] may also function for retargeting of UniCAR T cells against PCa tumor cells.

Over the past decade, highly promising antigen-specific immunotherapies were developed currently approaching the clinics which are based on either bispecific antibodies (bsAbs) or chimeric antigen receptors (CARs) [e.g. 5, 6]. Both strategies finally lead to a target-dependent antigen-specific cross-linkage between immune effector- and tumor cells as schematically shown in Figure 1. At the site of interaction, immune synapse-like structures are formed resulting in killing of tumor cells in a TCR and MHC independent manner [5–25] via the granzyme B/perforin pathway [16, 24, 25].

BsAbs can be generated by recombinant fusion of the variable domains of two monoclonal antibodies (mAbs): For retargeting of immune effector T cells, one antibody domain is directed to the activating CD3-complex, the other one to a tumor-associated surface antigen (TAA) [e.g. 6, 7]. The first clinically successfully applied BiTE (Bispecific T cell Engager) is the CD3xCD19 directed bsAb Blinatumomab [13]. Interestingly, CD19 directed CAR T cells can still efficiently kill Blinatumumab resistant tumor cells.

In principle, CARs represent genetically engineered synthetic receptors consisting of three portions: (i) an extracellular binding moiety, (ii) a transmembrane domain, and (iii) intracellular signaling domain(s). The extracellular antigen binding moiety is commonly a single-chain fragment variable (scFv) derived by recombinant fusion of the variable heavy and light chain sequences from a mAb. The transmembrane domain is commonly taken from the CD28- or CD8 receptor [e.g. 5]. The intracellular signaling domains are portions (ITAM motifs) of activating immune receptors. In dependence on the intracellular signaling domain(s) first, second and third generation CARs are differentiated [e.g. 5]. The tremendous clinical success of CD19-specific CAR T cells underlines the high potential of the CAR technology [26–29]. Nonetheless, there are still conceptual limitations inherent to this treatment strategy: Due to either inappropriate on target, off tumor reactions against healthy tissue or excessive on-target, on-tumor reactions against heavy tumor loads, dramatic adverse side effects can occur. As CD19 expression is not limited to leukemic cells but also found on healthy B cells, patients treated with CD19 CARs suffer from ongoing B cell aplasia. While the lack of B cells is manageable by intravenous immunoglobulin administration, in case of other TAAs such on-target, off-tumor effects may not be acceptable or may even become life-threatening [30, 31]. For example the prostate membrane specific antigen (PSMA) and the prostate stem cell antigen (PSCA) represent very promising tumor targets for PCa and also other carcinomas including for example pancreatic- and breast carcinomas [32–37]. Both targets are present on about 90% of human prostate tumors including metastases and their expression positively correlates with tumor stage [32–39]. However, both antigens are also expressed in healthy tissues: PSMA has been detected in salivary glands, brain, and kidneys [37–39]. Expression of PSCA was reported in bone, liver and lymph nodes [40]. Although CAR constructs directed to PSCA or PSMA were shown to be well functional *in vitro* and in mouse models [41–44], based on these expression data, an application of conventional CAR T cells may cause detrimental potentially life-threatening destruction of healthy tissues.

Recently we described a novel modular antibody based platform technology which may help to overcome such limitations [45, 46]. Originally, we separated the functional domains of a conventional BiTE onto two molecules as schematically summarized in Figure 1 (modular BiTE) [16, 18–21]. The two components were termed universal effector module (EM) and individual target module(s) (TM). The universal EM represents a bsAb: On the one hand it is directed to the activating CD3 complex of T cells, on the other hand, it is directed to a peptide epitope (E5B9) [e.g. 46, 47]. The interaction with the tumor cell is mediated via the TM. First TMs were scFvs directed to a tumor-associated antigen (TAA) to which the E5B9 epitope is fused. Thus, EM and TM can form an immune complex which works like a conventional bsAb (Figure 1) [16, 18, 46, 47]. In principle, the TMs could also be combined with a CAR directed against the same peptide epitope [45, 46]. We termed this CAR as universal CAR (UniCAR). UniCAR expressing T cells can reversibly be armed with one or even multiple TMs [45, 46, Bachmann unpublished]. Pharmakokinetic data show that recombinant antibody derivates such as scFvs are rapidly eliminated from peripheral blood. Therefore, we expect that UniCAR T cells will automatically be switched off when the respective TM is eliminated from a patient, thus providing a self limiting safety switch. For retargeting of T cells to PCa cells we recently described modular BiTEs to PSCA and PSMA leading to the question whether or not the same TMs may also work for retargeting of PCa cells with UniCAR T cells.

Indeed, here we show proof of concept for both *in vitro* and *in vivo* retargeting of PCa cells with UniCAR T cells armed with these TMs directed against either PSCA or PSMA or both TMs simultaneously.

## RESULTS

In previous studies we described TMs against PSCA and PSMA for use in our modular BiTE format (Figure 1) [16]. The TMs are based on well characterized mAbs directed to PSCA or PSMA [16, 17, 43]. In order to show that the same TMs may also work in combination with UniCAR T cells, the TMs were purified from cell culture supernatants of eucaryotic cells expressing the respective antibody derivates using Nickel affinity chromatography. Purified TMs were biochemically analyzed and characterized as described previously [e.g. 16] (see also MATERIALS AND METHODS).

For functional analysis, human T cells from healthy donors were transduced with lentiviral vectors encoding the UniCAR sequence containing a dual CD28/CD3ζ signaling domain (UniCAR 28/ζ). As negative controls, T cells were transduced with lentiviral vectors encoding the UniCAR sequence lacking the signaling domain (UniCAR Stop). As additional negative controls served either mock transduced T cells or T cells transduced with a vector encoding EGFP marker protein (vector control). In order to compare the efficacy of conventional CAR T cells with UniCAR T cells T cells were transduced with vectors encoding conventional CARs directed against PSCA or PSMA [41, 43]. In order to compensate different transduction rates of UniCAR positive T cells, the transduction efficacy was estimated by FACS analysis and transduced cells were sorted using another peptide epitope tag (7B6 [48]) which is part of the extracellular CAR domain [41]. Cells were sorted to >90% purity to allow comparison between different human donors. Transduction and sorting was performed as described previously [41] (see also MATERIALS AND METHODS).

### Activation of uniCAR T cells in a TM-dependent and target-specific manner

**Figure 2:**
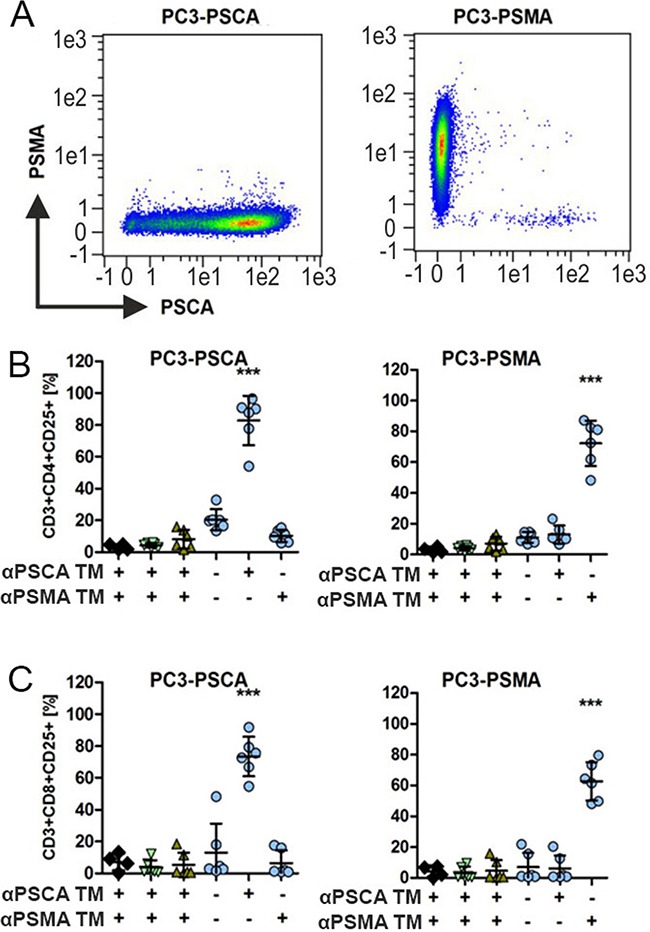
Activation of UniCAR T cells in dependence on PSCA or PSMA specific TMs **(A)** The prostate cell line PC3 down regulates the expression of both PSCA and PSMA. To overcome this limitation, PC3 cells were genetically engineered to reliably overexpress PSCA (left chart) or PSMA (right chart). Antigen expression was detected by FACS analysis using labelled mabs to PSCA or PSMA as described under MATERIALS AND METHODS. **(B, C)** Human T cells from healthy donors were mock transduced (rhombes) or transduced with lentiviral vectors encoding the UniCAR containing a dual CD28/CD3ζ signaling domain (UniCAR 28/ζ, circles) or lacking any signaling domain (head up triangle) or expressing only EGFP marker protein (head down triangle). UniCAR T cells were incubated with the genetically modified PC3 cells with 15 nM TMs specific for either PSCA (αPSCA TM) or PSMA (αPSMA TM). After 24h of cultivation, cells were harvested and stained for CD3, CD4, CD8 and CD25 surface expression as described in MATERIALS AND METHODS. Plots show the analysis of the activation marker CD25 for CD4 **(B)** and CD8 **(C)** T cells of six individual T cell donors (mean and s.d.). Statistical analysis was performed using non-parametric one-way ANOVA (Kruskal-Wallis test) and post-hoc Dunn's Multiple Comparison test (***p < 0.001).

For analysis of TM dependent and target specific activation of UniCAR T cells, we used PC3 cells expressing either PSCA (PC3-PSCA, Figure 2A) or PSMA (PC3-PSMA, Figure 2A) as confirmed by FACS analysis. The analysis of activation of UniCAR 28/ζ T cells is shown in (Figure 2B, 2C, circles). The data for the negative controls are shown in Figure 2B, 2C including for mock transduced T cells (Figure 2B, 2C, rhombes) or for UniCAR stop T cells (Figure 2B, 2C, head up triangle) or T cells expressing only EGFP vector control (Figure 2B, 2C, head down triangle). PC3-PSCA or PC3-PSMA cells were cocultured with or without such modified T cells either in the presence (Figure 2B, 2C, +) or absence (Figure 2B, 2C, −) of the respective TM (15 nM) against PSCA (Figure 2B, 2C, αPSCA TM) or PSMA (Figure 2B, 2C, αPSMA TM). After 24h of cultivation, cells were harvested and analyzed by FACS for CD3, CD4, CD8 and CD25 surface expression. Results for expression of the activation marker CD25 on CD4-positive UniCAR T cells are shown in Figure 2B. Results for expression of the activation marker CD25 on CD8-positive UniCAR T cells are shown in Figure 2C. As shown in Figure 2, both CD4- (Figure 2B) and CD8 (Figure 2C) UniCAR T cells were activated in a strictly TM-dependent and target-specific manner: UniCAR 28/ζ T cells were only activated by the combination PC3-PSCA and the PSCA specific TM (αPSCA TM, Figure 2B, 2C, left panels) but not in the presence of the PSMA specific TM (αPSMA TM, Figure 2B, 2C, left panels). Vice versa, UniCAR 28/ζ T cells were only activated by the combination PC3-PSMA and the PSMA specific TM (αPSMA TM, Figure 2B, 2C, right panels) but not in the presence of the PSCA specific TM (αPSCA TM, Figure 2B, 2C, right panels). As expected, in the absence of any TM UniCAR 28/ζ T cells are not activated on either PC3-PSCA (Figure 2C, left panels) or PC3-PSMA (Figure 2C, right panels). Moreover, all negative controls including mock transduced T cells (Figure 2B, 2C, rhombes), T cells expressing the UniCAR Stop sequence (Figure 2B, 2C, head up triangle), or T cells transduced with the vector control to express EGFP (Figure 2B, 2C, head down triangle) do not show any activation of modified T cells including in the presence of both TMs.

### Killing of PSCA and PSMA positive PCa cells by retargeted UniCAR T cells occurs in a TM-dependent and target-specific manner with an efficacy comparable to conventional CARs

**Figure 3:**
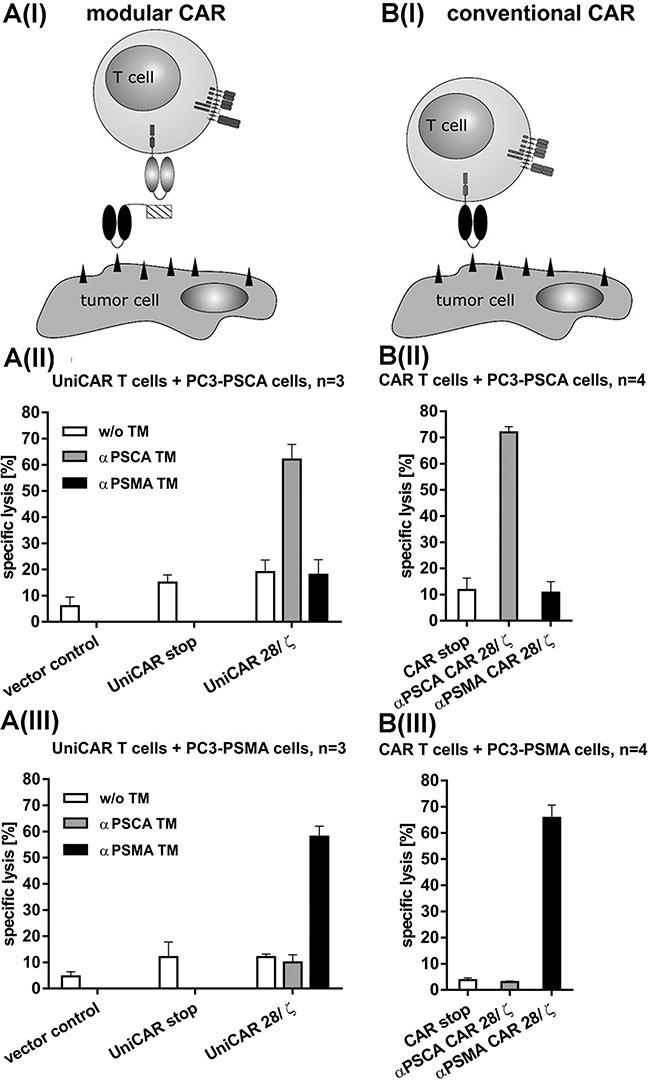
Retargeting of UniCAR- **(A)** and conventional **(B)** CAR T cells against PSCA or PSMA positive tumor cells. Schematic comparison of the UniCAR system **(A(I))** versus conventional CARs **(B(I))**. In order to compare the efficacy of UniCAR T cells with conventional CAR T cells the prostate cancer cell line PC3 was genetically engineered to overexpress either PSCA **(A(II), B(II))** or PSMA **(A(III), B(III))** as described under MATERIALS AND METHODS. **(A(II, III))** Human T cells were transduced with lentiviral vectors encoding the UniCAR containing a dual CD28/CD3ζ signaling domain (UniCAR 28/ζ) or lacking any signaling domain (UniCAR stop) or expressing only EGFP marker protein (Vector control). The respective UniCAR T cells were armed with TMs (1 nM) directed to either PSCA (αPSCA TM, grey bars) or PSMA (αPSMA TM, black bars) or incubated in the absence of any TM (w/o TM, white bars). **(B(II, III))** Human T cells were transduced with lentiviral vectors encoding conventional CARs containing a dual CD28/CD3ζ signaling domain. The CARs were directed against either PSCA (αPSCA CAR 28/ζ, grey bars) or PSMA (αPSMA CAR 28/ζ, black bars). Alternatively, the human T cells were transduced with the respective (αPSCA or αPSMA) conventional CAR construct but lacking any signaling domain (CAR stop, white bars). Mean and s.d. from experiments with three (UniCAR) or four (conventional CARs) individual T cell donors is shown.

For killing analysis we performed chromium release assays (see MATERIALS AND METHODS) using PSCA- (Figure 3A(II), B(II)) or PSMA- (Figure 3A(III), B(III)) overexpressing PC3 cells. T cells were transduced with either UniCARs (Figure 3A(I-III)) or conventional CARs (Figure 3B(I-III)). Chromium release was measured after a 24h incubation at an effector to target cell (e:t) ratio of 5 to 1 (Figure 3A(II, III), B(II, III)). As shown in Figure 3, both, UniCAR 28/ζ and conventional CAR T cells were able to kill the target cells at comparable rates. The killing of the target cells via UniCAR T cells was strictly dependent on the presence of the respective TM (1 nM) either αPSCA TM (Figure 3(A II)) or αPSMA TM (Figure 3(A III)) and occurred in a target-specific manner. No killing of PSCA-positive PC3 cells was observed with UniCAR 28/ζ T cells in the presence of the PSMA TM (Figure 3(A II)) and vice versa of PSMA-positive PC3 cells in the presence of the PSCA TM (Figure 3(A III)). Furthermore, no killing was observed with UniCAR 28/ζ T cells in the absence of any TM (Figure 3(A II, III)). Moreover, T cells modified with the UniCAR stop vector or the EGFP encoding vector control did not attack the target cells (Figure 3(A II, III)). In agreement with previous studies [41, 43], the conventional CAR T cells directed to PSCA only attacked PSCA-positive PC3 cells but not PSMA-positive PC3 cells (Figure 3(B II)) and vice versa the conventional CAR T cells directed to PSMA only attacked PSMA-positive PC3 cells but not PSCA-positive PC3 cells (Figure 3(B III)). Furthermore, T cells modified with the respective conventional stop vector CAR construct did not attack target cells (Figure 3(BII, III)).

### Release of cytokines by retargeted uniCAR T cells occurs in a TM- and target-specific manner

**Figure 4:**
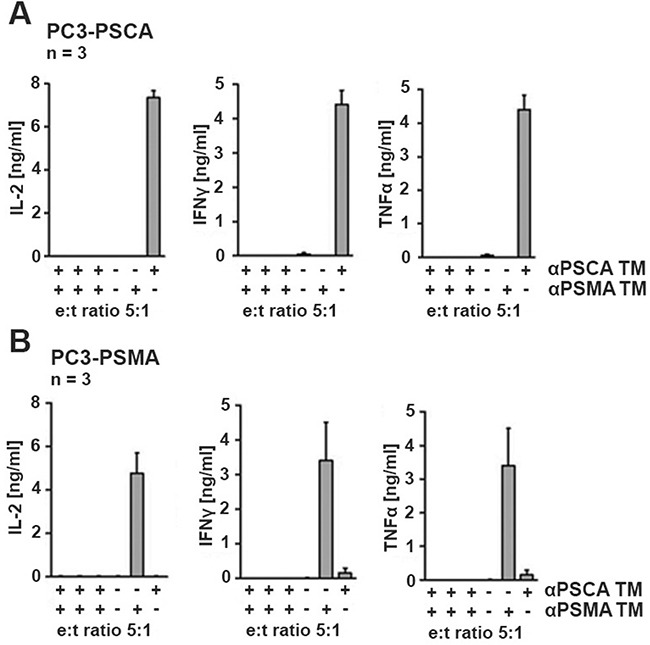
Analysis of cytokine release from UniCAR T cells UniCAR 28/ζ T cells were incubated with **(A)** the genetically modified PC3-PSCA cells with 15 nM TM specific for PSCA (αPSCA TM) or **(B)** the genetically modified PC3-PSMA cells with 15 nM TM specific for PSMA (αPSMA TM). After 24h of cultivation, cell-free culture supernatants were analyzed by ELISA for release of IFNγ, IL-2, IL-6 and TNFα. IL-6 could not be detected (data not shown, see also Figure 9). As negative controls (left to right) served mock transduced T cells, T cells expressing the UniCAR stop sequence, or T cells transduced with the vector control to express EGFP. All these controls were analyzed in the presence (+) of both TMs. As additional negative control, supernatants from assays containing UniCAR 28/ζ T cells but in the absence (−) of any TM were analyzed.

For analysis of cytokine release, UniCAR 28/ζ T cells were incubated in the presence (Figure 4, +) or absence (Figure 4, −) of either the TM (15 nM) αPSCA TM or αPSMA TM or the absence of both TMs. The modified T cells were incubated with either PC3-PSCA cells (Figure 4A) or PC3-PSMA cells (Figure 4B). After 24h of cultivation, cell-free culture supernatants were analyzed by ELISA for release of IFNγ, IL-2, TNFα, and IL-6. As negative controls (Figure 4A, 4B, in each chart left to right) served T cells expressing the UniCAR stop sequence, or T cells transduced with the vector control to express EGFP. IL-6 was not released at measurable amounts at all (data not shown, see also Figure 9). The cytokines IFNγ, IL-2, and TNFα were detected but only in supernatants of the UniCAR 28/ζ T cells. Cytokine release was strictly dependent on combination of TM and target cell: Cytokines were only released when UniCAR 28/ζ T cells were retargeted to PSCA positive cells via the αPSCA TM but not via the αPSMA TM (Figure 4A). And vice versa, cytokines were only released from UniCAR 28/ζ T cells when retargeted to PSMA positive cells via the αPSMA TM but not via the αPSCA TM (Figure 4B). No cytokine release was observed with UniCAR 28/ζ T cells in the absence of any TM. Cytokines could also not be detected in supernatants of any negative control (Figure 4A, 4B).

### Estimation of range of working concentrations for UniCAR T cells armed with an αPSCA- and αPSMA TM

**Figure 5:**
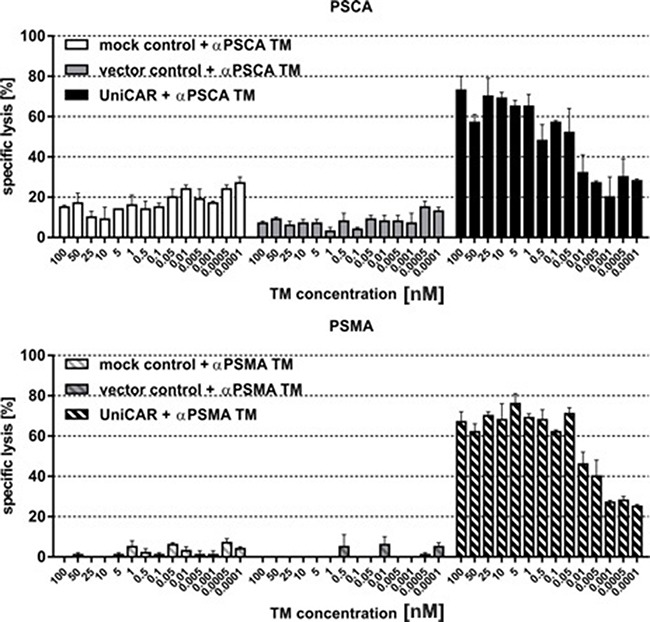
Estimation of range of working concentrations for αPSCA- and αPSMA TM UniCAR T cells or controls (mock, vector control) were incubated with [^51^Cr]-loaded **(A)** PSCA- or **(B)** PSMA overexpressing PC3 cells at an effector to target cell (e:t) ratio of 5:1. The respective TM was added at the indicated concentrations. After 20h of cultivation, target cell lysis ([^51^Cr]-release) was measured. Data for a single T cell donor is shown.

Next we wanted to show that the lysis of tumor cells via UniCAR T cells also occurs in a TM-dependent and target-specific manner at low and high concentrations of TMs. For that purpose lysis of PC3-PSCA (Figure 5A) and PC3-PSMA cells (Figure 5B) were analyzed by titration experiments. UniCAR 28/ζ T cells (Figure 5A, 5B, right panels) or negative controls (mock, Figure 5A, 5B left panels, vector control, Figure 5A, 5B, middle panels) were incubated with [^51^Cr]-loaded PC3-PSCA (Figure 5A) or PC3-PSMA cells (Figure 5B) at an effector to target cell (e:t) ratio of 5 to 1. The respective TM (Figure 5A, αPSCA TM or Figure 5B, αPSMA TM) was added at the indicated concentrations. After 20h of cultivation, target cell lysis ([^51^Cr]-release) was measured. These titration experiments revealed that a TM dependent PC3 cell lysis was even seen at as low as 0.05 nM. The different background lysis seen in Figure 5 is caused by donor dependent variations.

### Comparison of the UniCAR- with the modular BiTE format

**Figure 6:**
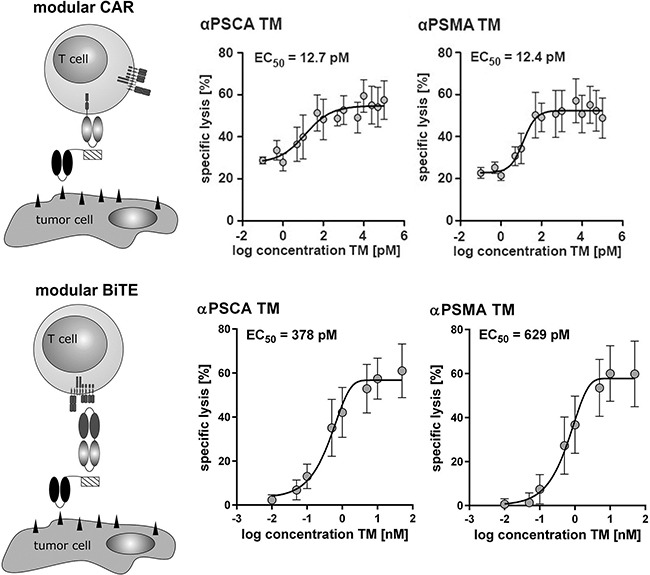
Comparison of the UniCAR- with the Modular BiTE Format For estimation of the respective EC_50_ values αPSCA- or αPSMA TM were combined with either UniCARs (upper panel) or the bispecific effector module (CD3xE5B9) (lower panel) and unmodified T cells. The respective T cells were incubated with [^51^Cr]-loaded PC3 cells at an effector to target cell (e:t) ratio of 5:1. After 20h of cultivation, cell lysis ([^51^Cr]-release) was measured. Plots show mean and s.d. from experiments for three individual T cell donors.

Further titration experiments allowed us to calculate an EC_50_ value of 12.7 pM for the αPSCA TM and an EC_50_ value of 12.4 pM for the αPSMA TM when used in combination with UniCAR T cells (Figure 6, upper panel). As schematically summarized in Figure 6 (modular BiTE, lower panel) the same TMs can be combined with a common bispecific EM (effector module, see also Figure 1) for retargeting of unmodified T cells to tumor cells. In order to compare the efficacy of the UniCAR- with the modular BiTE system, similar titration experiments were performed for such a modular BiTE complex (Figure 6, lower panel). For the modular BiTE format we calculated EC_50_ values of 378 pM for the αPSCA TM and 629 pM for the αPSMA TM, respectively (Figure 6, lower panel).

### Retargeting of UniCAR T cells to tumor cells (over) expressing the TAA either naturally or artificially

**Figure 7:**
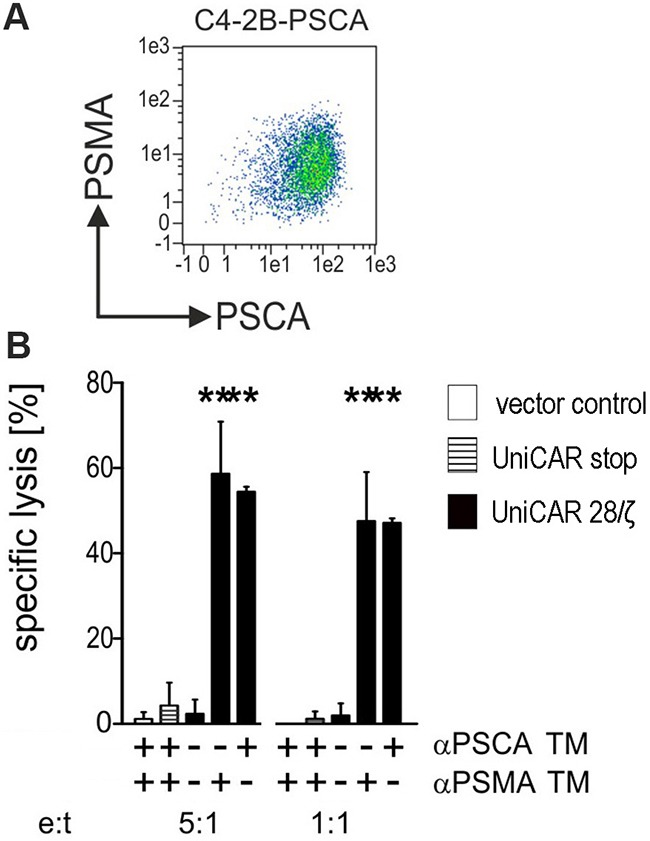
Retargeting of UniCAR T cells against tumor cells which naturally express PSMA and artificially overexpress PSCA **(A)** The prostate cell line LNCaP naturally expresses PSMA but like other PCa cells downregulates PSCA in cell culture. In order to reliably express both PSCA and PSMA LNCaP cells were genetically engineered to express PSCA in addition to the endogenous PSMA resulting in the LNCaP sub-line C4-2B-PSCA. Antigen expression was detected by FACS using mabs as described under MATERIALS AND METHODS. **(B)** Human T cells were transduced with lentiviral vectors encoding the UniCAR containing a dual CD28/CD3ζ signaling domain (UniCAR 28/ζ, black bars) or lacking any signaling domain (UniCAR stop, stripped bars) or expressing only EGFP marker protein (vector control, open bars). UniCAR T cells were armed with TMs directed to either PSCA (αPSCA TM) or PSMA (αPSMA TM) and incubated with [^51^Cr]-labelled C4-2B-PSCA cells at e:t ratios of 5:1 and 1:1. UniCAR armed T cells killed antigen double-positive LNCaP-C4-2B only in the presence of either αPSCA- or αPSMA specific TMs (1 nM). [^51^Cr]-release was estimated after a 24h incubation. Mean and s.d. from experiments with three individual T cell donors is shown. Statistical analysis was performed using non-parametric one-way ANOVA (Kruskal-Wallis test) and post-hoc Dunn's Multiple Comparison test (***p < 0.001).

So far, UniCAR T cells were retargeted to PC3 cells artificially overexpressing either PSCA or PSMA or both. According to our own experience all so far analyzed PCa cell lines downregulate PSCA under cell culture conditions [16]. The only cell line known to us expressing PSCA in cell culture is the bladder carcinoma cell line RT4 which does not express PSMA. In contrast, the PCa cell line LNCaP naturally expresses PSMA but like the other PCa lines also down regulates the expression of PSCA. In order to achieve a reliable expression of PSCA, LNCaP cells were transduced to overexpress PSCA. As shown in Figure 7(A) the resulting LNCaP-C4-2B subline expresses both PSMA naturally and PSCA artificially as analyzed by FACS. Chromium release was performed after a 24h incubation at the indicated effector to target cell (e:t) ratios of 5 to 1 and 1 to 1 (Figure 7B). As shown in Figure 7, only UniCAR 28/ζ T cells were able to kill the target cells. Moreover, the killing of the target cells was strictly dependent on the presence (Figure 7B, +) of the TM (1 nM) either αPSCA TM or αPSMA TM. No killing was observed with UniCAR 28/ζ T cells in the absence (Figure 7B, −) of any TM. Moreover, T cells modified with the UniCAR Stop vector or the EGFP encoding vector control did not attack the target cells (Figure 7B).

### Simultaneous targeting of PSCA- and PSMA positive tumor cells with UniCAR T cells armed with both αPSCA- and αPSMA TMs

**Figure 8:**
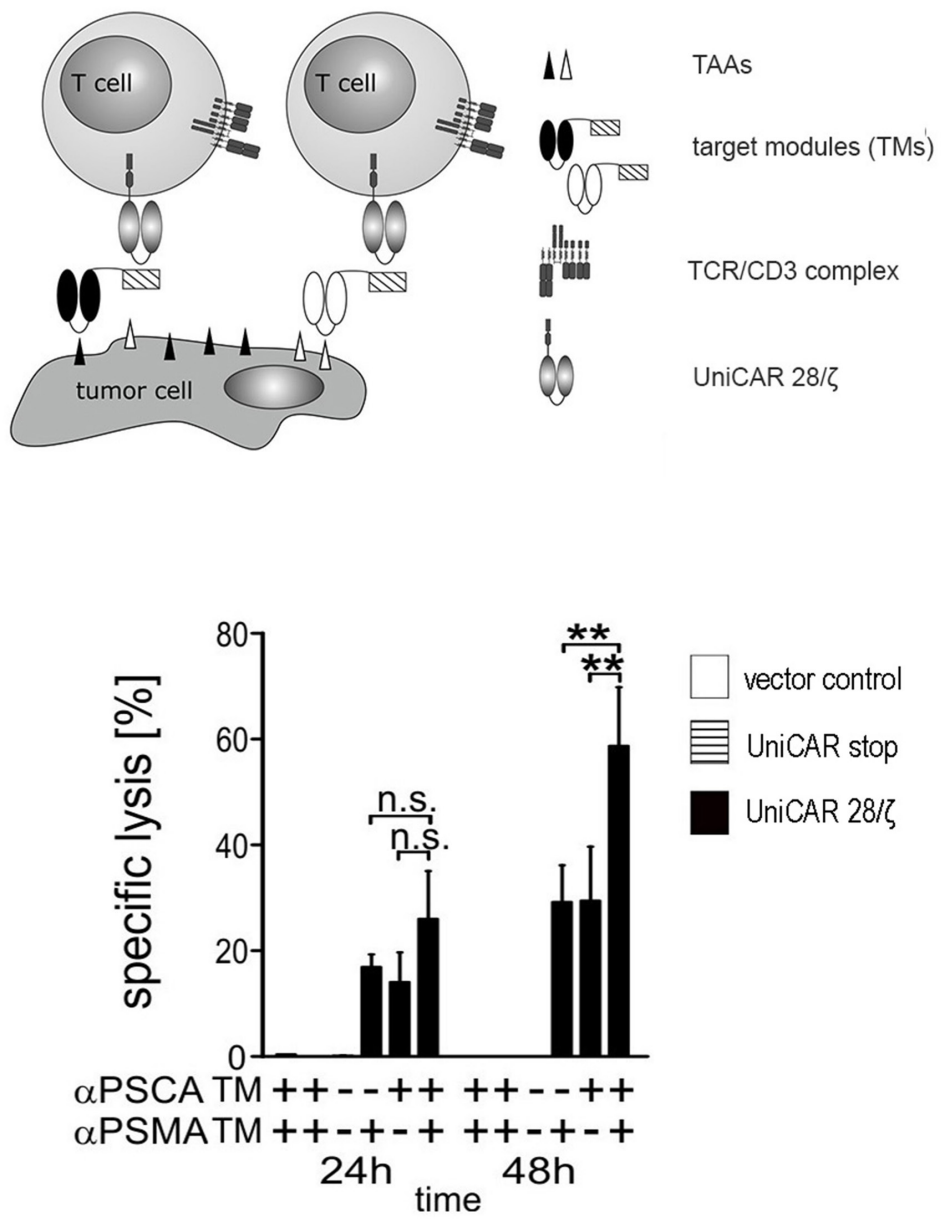
Simultaneous targeting of PSCA- and PSMA positive tumor cells with UniCAR T cells armed with both αPSCA- and αPSMA TMs Human T cells were transduced with lentiviral vectors encoding the UniCAR containing the dual CD28/CD3ζ signaling domain (UniCAR 28/ζ T cells, see also Figure 3). UniCAR T cells were incubated with PSCA and PSMA double-positive PC3 cells at an effector to target cell (e:t) ratio of 1:2 with either 1 nM of αPSCA TM οr 1 nM of αPSMA TM or combining both TMs at a total concentration of 1 nM. Specific lysis was estimated by [^51^Cr]-release after 24h or 48h, respectively. Mean and s.d. from experiments with three individual T cell donors is shown. Statistical analysis were performed using non-parametric one-way ANOVA (Kruskal-Wallis test) and post-hoc Dunn's Multiple Comparison test (ns = not significant, **p < 0.01).

One of the unique advantages of the UniCAR system is the possibility to redirect CAR T cells to more than one target either sequentially or simultaneously. For simultaneous retargeting PC3 cells overexpressing both PSCA and PSMA were used as target cells. In principle, the assay as described in Figure 3 was repeated but using a lower effector- to target cell (e:t) ratio of 1 to 2 with either 1 nM of αPSCA TM οr 1 nM of αPSMA TM or combining both TMs at a total concentration of 1 nM. The results obtained in the presence (Figure 8, +) or absence (Figure 8, −) of the respective TM are given in Figure 8. Specific lysis was estimated by [^51^Cr]-release after 24h or 48h, respectively. As shown in Figure 8, the tumor cell lysis occurs significantly faster (**p < 0.01) when UniCAR 28/ζ T cells are simultaneously armed with both the αPSCA- and the αPSMA TM. In line with the data shown in Figure 3, no killing was observed in the respective negative controls (Figure 8, 24h and 48h, left to right) including in the presence of both TMs by T cells expressing the UniCAR Stop vector or the EGFP encoding vector, or in the absence of both TMs by UniCAR 28/ζ T cells (Figure 8).

**Figure 9:**
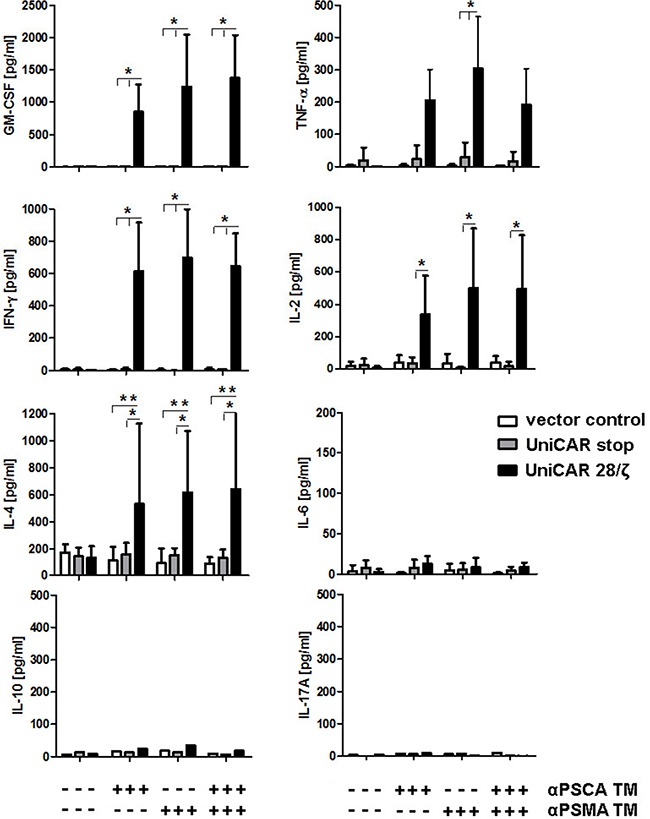
Mulitplex analysis of cytokine release from UniCAR T cells: Comparison of mono- versus dual-targeting UniCAR 28/ζ T cells were incubated with PC3 cells which were genetically modified to overexpress both PSCA and PSMA either in the presence (+) or absence (−) of TMs specific for either PSCA (αPSCA TM, 1 nM), PSMA (αPSCA TM, 1 nM) or both (0.5 mM each). For negative controls T cells were transduced with either the vector control encoding EGFP (vector control) or UniCARs lacking intracellular signaling domains (UniCAR stop). After 24h of cultivation, cell-free culture supernatants were analyzed using the MACSPlex Cytokine 12 Kit (see MATERIALS AND METHODS). This bead-based multiplex assay allowed us to detect and quantify the cytokines GM-CSF, IFN-α, IFN-γ, IL-2, IL-4, IL-5, IL-6, IL-9, IL-10, IL-12, IL-17A and TNF-α in parallel. Selected data for four donors are shown. All cytokines not shown were negative. With the exception of IL-10 and IL-17A which could only be detected in samples from one of the four analyzed donors, the mean and s.d. from experiments for four individual T cell donors is shown. Statistical analysis was performed using non-parametric one-way ANOVA (Kruskal-Wallis test) and post-hoc Dunn's Multiple Comparison test (ns = not significant, *p < 0.1, **p < 0.01).

In addition, we estimated the cytokines released using a multiplex assay - the MACSPlex Cytokine 12 Kit - instead of ELISA. This bead-based assay allowed us to detect and quantify the cytokines GM-CSF, IFN-α, IFN-γ, IL-2, IL-4, IL-5, IL-6, IL-9, IL-10, IL-12, IL-17A and TNF-α in parallel. Selected data for four donors analyzed are shown in Figure 9. With the exceptions of GM-CSF, IFN-γ, IL-2, IL-4, and TNF-α (Figure 9) no other cytokines could be detected at a significant concentration including IL-6. Cytokines were only detected in samples from UniCAR expressing T cells in the presence of (i) target cells and (ii) at least one of the TMs (Figure 9 +). The amount of the respective cytokine estimated in the presence of both, the αPSCA- and αPSMA TM (0.5 nM each) did not differ from the amount of cytokine that was measured in the presence of just one of the TMs (1 nM). Cytokines could not be detected in supernatants of any of the negative controls (Figure 9, UniCAR T cells in the absence of a TM -). In case of IL-10 and IL-17A only low levels of cytokines were detected and only for one of the four donors.

### The UniCAR system triggers anti-tumor effects in experimental mice in dependence on the presence of TMs

**Figure 10:**
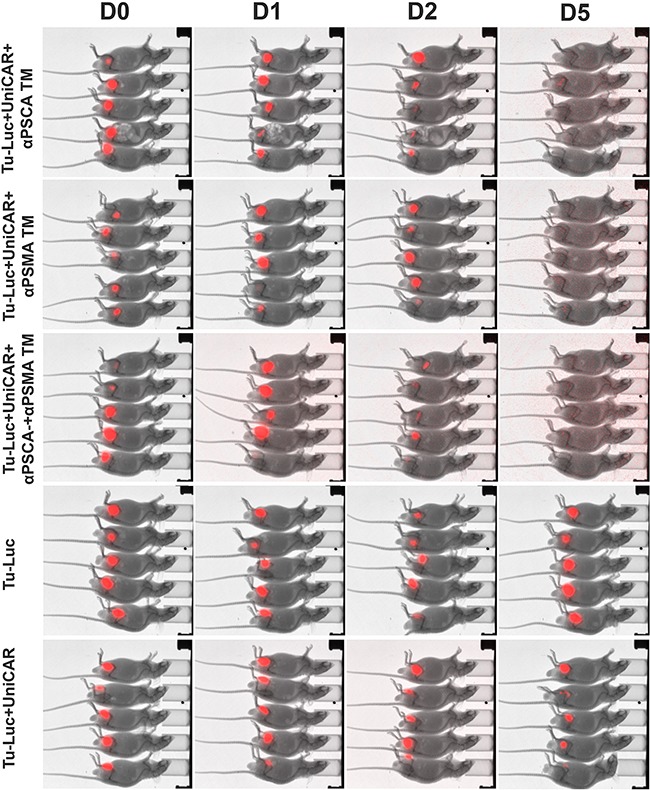
Retargeting of PSCA and PSMA positive tumor cells in experimental mice PC3 cells were transduced to simultaneously overexpress PSCA, PSMA and firefly luciferase (see MATERIALS AND METHODS) resulting in triple positive PC3 cells termed Tu-Luc. 1×10^6^ Tu-Luc cells were mixed with 0.5×10^6^ UniCAR 28/ζ T cells and 300 pM per mouse of the respective TM including the αPSCA TM (Tu-Luc+UniCAR+αPSCA TM) or αPSMA TM (Tu-Luc+UniCAR+αPSMA TM) or both simultaneously (Tu-Luc+UniCAR+αPSCA TM+αPSMA TM). As “untreated” controls served either 1×10^6^ Tu-Luc cells alone (Tu-Luc) or Tu-Luc cells were mixed with 0.5×10^6^ UniCAR 28/ζ T cells without any TM (Tu-Luc+UniCAR). The respective mixture (100 μl) was injected subcutaneously into female NMRI-Foxn1nu/Foxn1nu mice resulting in five groups of animals each consisting of five mice. Luminescence imaging of anesthesized mice was performed 10 min after i.p. injection of 200 μl of luciferin (15 mg/ml) starting at day zero (D0), followed at day one (D1), day two (D2), and day 5 (D5).

In order to show a TM-dependent *in vivo* anti-tumor activity of UniCAR T cells against PCa cells, a novel mouse model was established. For this purpose, PC3 cells were transduced to simultaneously overexpress PSCA, PSMA and firefly luciferase (see MATERIALS AND METHODS). The triple positive PC3 cells were termed Tu-Luc (Figure 10). 1×10^6^ Tu-Luc cells were mixed with 0.5×10^6^ UniCAR 28/ζ T cells and 300 pM per mouse of the respective TM including the αPSCA TM (Figure 10, Tu-Luc+UniCAR+αPSCA TM) or αPSMA TM (Figure 10, Tu-Luc+UniCAR+αPSMA TM) or both simultaneously (Figure 10, Tu-Luc+UniCAR+αPSCA TM+αPSMA TM). As “untreated” controls served either 1×10^6^ Tu-Luc cells alone (Figure 10, Tu-Luc) or mixed with 0.5×10^6^ UniCAR 28/ζ T cells without any TM (Figure 10, Tu-Luc+UniCAR). The respective mixture (100 μl) was injected subcutaneously into female NMRI-Foxn1nu/Foxn1nu mice resulting in five groups of animals each consisting of five mice (Figure 10). Each group of mice was analyzed in parallel for luciferase activity starting at day zero (Figure 10, D0), followed at day one (Figure 10, D1), day two (Figure 10, D2), and day 5 (Figure 10, D5). As shown in Figure 10, at day 5, no more luciferase activity can be detected in all the fifteen treated animals while luciferase activity can easily be detected in all the ten control mice. These data indicate that UniCAR T cells armed with TMs specific for PSCA or PSMA can also eliminate PSCA- and PSMA positive tumor cells *in vivo*.

## DISCUSSION

As next generation of bsAbs we recently introduced a novel modular BiTE platform that is universally applicable for redirection of T lymphocytes to different TAAs [16, 18]. Side by side comparison showed that modular BiTEs work as well as conventional BiTEs without any structural optimization [7, 15, 16]. T cells equipped with CARs appear to be even more efficient than BiTEs as patients resistant to Blinatumumab still responded to CD19 CAR T cells. One major draw back of currently used CARs is their risk of severe adverse effects which can occur during or even after tumor cell elimination. In case of life-threatening side effects the adoptively transferred CAR T cells have to be stopped as fast as possible e.g. via a suicide gene or by application of an antibody against the extracellular domain of the CAR [e.g. 49]. In order to avoid an elimination of the genetically modified CAR T cells we recently introduced a modular CAR platform termed UniCAR [46]: UniCAR equipped T cells are not directed to a cell surface target and therefore are inert. They become active only in the presence of a TM. Bearing in mind that TMs based on e.g. scFvs are rapidly eliminated we expect that UniCAR T cell activity can be titrated in patients in a dose-dependent manner. In case patients treated with UniCAR T cells suffer from tumor lysis- or cytokine release syndrome or after all the tumor cells have been eliminated UniCAR T cells can be turned off by stopping the infusion of the TM. If the tumor recovers, UniCAR T cells can be restarted. If tumor cells resistant to the applied TM evolve during therapy, a TM with another specificity should help to overcome this problem. In order to reduce the risk of the occurrence of tumor escape variants, a simultaneous application of two or even more TMs would also be possible. When applying a novel TM with potentially unknown side effects, the chance to titrate the CAR response by dosing the TM should increase the safety of the therapy. If tumor cells upregulate resistant factors such as check point inhibitors, TMs co-delivering stimulatory signals could help to circumvent this problem [16, 18].

Previously we described PSCA- and PSMA TMs [16]. In combination with the universal bispecific EM these TMs formed a bispecific immune complex (modular BiTE) which efficiently redirected unmodified T cells to PCa tumor cells [16]. It remained, however, unclear whether or not the same TMs would also work in combination with UniCAR T cells. Indeed, here we present experimental evidence that retargeting of UniCAR T cells via either the PSCA- or PSMA specific TM or both TMs simultaneously mediates the lysis of PSCA- and/or PSMA expressing PCa cell lines in a strictly target dependent- and target-specific, but MHC- and TCR-independent manner. Neither one of the TMs nor the signaling UniCARs alone caused tumor cell lysis. Lysis of tumor cells was not limited to artificially TAA overexpressing tumor cells as also naturally PSMA expressing tumor cells were eliminated. In agreement with previously published data [17], both polyclonal CD4^+^ and CD8^+^ T cells were activated upon target-dependent- and target-specific cross-linkage with target cells. Activation of UniCAR T cells resulted in the release of the cytokines. Cytokine release was estimated by MACSPlex assay and confirmed by ELISA. Activated UniCAR T cells released the cytokines GM-CSF, IL-2, IL-4, TNF and IFN-γ. Release of IL-6 could not be detected by either ELISA or the MACSPlex assay. The data obtained with the MACSPlex assay were comparable with the ELISA data for the cytokines IFN-γ and TNF-α. However, the IL-2 values estimated with the MACSPlex assay were significantly lower compared to the ELISA data. The reason is not clear yet. We have observed similar discrepancies between IL-2 ELISA and the MACSPlex assay when released cytokines were analyzed including for example for T cells redirected to different tumor cells via different bispecific abs (Bachmann, unpublished). Therefore, the MACSPlex assay may be less sensitive for IL-2 compared to the IL-2 ELISA.

Finally, the TM-dependent killing capability of UniCAR T cells armed with either the TM against PSCA or PSMA or both was also confirmed in a xenografted tumor mouse model for metastatic disease.

Taken together, both TM armed UniCAR T cells killed the respective target cells equally to conventional CAR T cells. According to the estimated EC_50_ values UniCARs should be more efficient than the modular BiTE complexes. Bearing in mind that modular BiTEs were as efficient as conventional BiTEs [16] our data support a ranking: UniCAR=CAR>BiTE=modular BiTE.

In summary, our data show that UniCAR T cells can efficiently be armed with TMs against the prostate tumor targets PSCA and PSMA. Retargeting of such armed UniCAR T cells to PCa cells expressing either PSCA or PSMA or both targets results in an efficient elimination of the tumor cells both *in vitro* and *in vivo*. Due to the self regulatory capability, the UniCAR system represents a promising platform for retargeting of tumor cells including PCa cells using otherwise critical surface targets such as PSMA and PSCA.

## MATERIALS AND METHODS

### Cell lines

The prostate cancer cell line PC3, LNCaP as well as the CHO cell line were purchased from American Type Culture Collection and have not been further authenticated. By FACS analysis an expression of PSCA was not detected in PC3 or LNCaP. PSMA expression was detectable in LNCaP but also not in PC3 cells. Therefore, PC3 cells were transduced with the open reading frame (orf) encoding PSCA or PSMA or both. LNCaP cells were transduced with orf encoding PSCA. For *in vivo* analysis the PC3 cell line expressing PSCA and PSMA was further modified to express the gene encoding firefly luciferase (Tu-Luc). Transduction was performed using a lentiviral packaging system as described previously [e.g. 15]. All cell lines were cultured in RPMI 1640 medium completed with 10% FCS, 100 U/ml penicillin and 100 μg/ml streptomycin, 2 mM N-acetyl-L-alanyl-L-glutamine, 1% non-essential amino acids and 1 mM sodium pyruvate (Biochrom). Cells were maintained at 37 °C in a humidified atmosphere of 5 % CO_2_.

### Construction and expression of recombinant antibodies

Cloning of the TMs against PSCA and PSMA into the lentiviral vector p6NST50 was described previously [16]. Stable recombinant TM producing CHO cell lines were established and recombinant proteins were purified from cell culture supernatants via Ni-NTA affinity chromatography followed by analysis of protein concentration and purity through SDS-PAGE and immunoblotting as described [16, 17, 50].

### Generation of UniCAR vectors

The cloning of the humanized anti-La 5B9 single chain fragment variable [18, 46], generation of the hinge, transmembrane and signaling domain of the UniCAR was recently described in detail [46]. The scFv was fused at the 5′ end of the CD28 coding sequence with a peptide epitope of 18 aa (7B6-tag) [41, 48] and an additional G_4_S_1_ linker in between the scFv and CD28. The monoclonal antibody 7B6 was generated by standard hybridoma fusion technique and identified to be reactive against the introduced epitope sequence [48]. Both epitopes are parts of the nuclear autoantigen La/SS-B [51]. The CAR signaling and stop constructs were subsequently cloned into the lentiviral vector p6NST60 [48]. The orf of the CAR constructs were fused to an EGFP orf separated by a 2pA protease site derived from the *Thosea asigna* virus, which allows an independent translation of CAR and EGFP from a single mRNA in modified T cells [52].

### Isolation of peripheral blood mononuclear cells (PBMCs), T cell subpopulations and lentiviral transduction

Isolation of primary human T cells from peripheral blood mononucleated cells (PBMCs) out of buffy coats (supplied by German Red Cross, Dresden, Germany), from fresh blood, or apheresis products of healthy donors and patients was performed as described [17]. The study including the consent form was approved by the local ethics committee of the university hospital of the medical faculty of Carl-Gustav-Carus TU-Dresden (EK27022006). Isolated T cells were cultured in RPMI 1640 complete medium supplemented with 200 U/ml IL-2 (Proleukin® S, Novartis Pharmaceuticals, Horsham, UK), 5 ng/ml IL-7 and 5 ng/ml IL-15 (ImmunoTools, Friesoythe, Germany) at densities of 1-2×10^6^ cells/ml. Production of lentiviral particles and transduction of primary human T cells was carried out as described before [41]. Briefly, T cells were activated with anti-CD3/CD28 coated polyclonal T cell activator beads (ThermoFisher) at a bead to cell ratio of 1:4 for 24h and concentrated lentiviral vector supernatant was added five times for the next 48h at a multiplicity of infection of 10-15 in total before beads were removed. After successful genetic modification, T cells were maintained in RPMI supplemented with cytokines and three to four days later purified by cell sorting using a FACSAria II (BD Biosciences). After purification T cells were rested in RPMI supplemented with cytokines for additional 5-6 days. Media was substituted for complete RPMI lacking any recombinant cytokines 24h before experiments were performed as described [41].

### T cell activation and cytokine-release assay

For activation experiments, 1×10^5^ gene modified T cells were seeded in 96-well plates in triplets together with target cells. TMs were added at the indicated concentrations. After a 48h- cultivation CD25 surface expression on T cells was analyzed using a MACSQuant Analyzer®. Cell free supernatants were harvested after 24h from cultures to determine cytokine concentrations by using OptEIA™ Human IFN-γ, OptEIA™ Human IL-2, OptEIA™ Human IL-6, and OptEIA™ Human TNF ELISA Kits (BD Biosciences, Heidelberg, Germany). Alternatively, cell culture supernatants were analyzed using the MACSPlex Cytokine 12 Kit (Miltenyi Biotec GmbH), a MACSQuant^®^ Analyzer (Miltenyi Biotec GmbH) and the MACSQuantify^®^ software (Miltenyi Biotec GmbH) according to the manufacturer‘s instructions.

### Flow-cytometry analysis

Isolated T cells were stained with fluorochrome-labeled mabs directed against human CD4/VioBlue (Miltenyi Biotec, clone VIT4), CD3/PE-Cy7 (Biolegend, San Diego, USA, clone UCHT1), CD8/APC (BD Bioscience, clone RPA-T8), and CD25/PE (MiltenyiBiotec, clone 4E3). For detection of CAR surface expression T cells were incubated with mab 7B6 and subsequently stained with PE-labeled goat anti-mouse IgG (Beckmann Coulter, Krefeld, Germany). Samples were analyzed using a MACSQuant® Analyzer and MACSQuantify® software (Miltenyi Biotec) [47].

### T cell killing assay

For lysis of tumor cells standard chromium release assays were performed as described [e.g. 17].

### Optical imaging of tumor xenograft models

All animal experiments were carried out at the Helmholtz Zentrum Dresden Rossendorf (HZDR) according to the guidelines of German Regulations for Animal Welfare and have been approved by the Landesdirektion Dresden (24-9165.40-4, 24.9168.21-4/2004-1). Four weeks old female NMRI-Foxn1nu/Foxn1nu mice were purchased from JANVIER LABS (St. Berthevin, France). General anesthesia was induced with 10% (v/v) and maintained with inhalation of 8% (v/v) desflurane (Suprane, Baxter, Germany) in 30/10% (v/v) oxygen/air. Luminescence imaging (exposure times 1 s, 10 s, and 60 s) was performed using a dedicated small animal multimodal imaging system (Xtreme, Bruker, Germany) 10 min after i.p. injection of 200 μl of luciferin (15 mg/ml) (Thermofisher, Dreieich, Germany). In parallel an X-RAY photograph was taken from the same animals at the same position.

### Statistical analysis

Statistical analysis was performed with GrapPad Prism software version 5.0 (GraphPad Software Inc., La Jolla, CA, USA).
